# Characterization of *Salmonella* spp. Isolates from Swine: Virulence and Antimicrobial Resistance

**DOI:** 10.3390/ani10122418

**Published:** 2020-12-17

**Authors:** Hai Nguyen Thi, Thi-Thanh-Thao Pham, Barbara Turchi, Filippo Fratini, Valentina Virginia Ebani, Domenico Cerri, Fabrizio Bertelloni

**Affiliations:** 1 Department of Animal Science, Hong Duc University, Thanh Hoa City 40000, Vietnam; nlhaitd@gmail.com; 2 Biology Faculty, Dalat University, 01 Phu Dong Thien Vuong, Da Lat, Lam Dong 66000, Vietnam; thaoptt@dlu.edu.vn; 3 Department of Veterinary Science, University of Pisa, Viale delle Piagge 2, 56124 Pisa, Italy; barbara.turchi@unipi.it (B.T.); filippo.fratini@unipi.it (F.F.); valentina.virginia.ebani@unipi.it (V.V.E.); domenico.cerri@unipi.it (D.C.)

**Keywords:** *Salmonella*, swine, gastric acid resistance, virulence genes, antimicrobial resistance

## Abstract

**Simple Summary:**

*Salmonella* is a pathogenic bacterium able to infect both humans and animals. It is diffused worldwide and, generally, animals are a source of infection for humans. Among domestic animals, swine represents an important reservoir and a frequent source of human infection, especially in some countries like Italy. To acquire information on *Salmonella*, in particular about epidemiology, but also virulence, pathogenesis and antimicrobial resistance, is the basis for a cohesive control program. This manuscript describes an investigation conducted on *Salmonella* isolates from swine, where two important characteristics were evaluated: the pathogenicity and the antimicrobial resistance. A great variability was observed among investigated strains. *Salmonella* serovar Typhimurium was confirmed as one of the most virulent serovars; indeed, most isolates belonging to this serovar presented many of the searched virulence factors. A high level of antimicrobial resistance was observed for some compounds (sulfonamide, tetracycline, streptomycin and ampicillin), but not for the so-called “last line antibiotics”, such as, for example, ciprofloxacin. The constant monitoring on circulating strains in reservoir animals is important to acquire information and set up adequate prophylaxis measures.

**Abstract:**

*Salmonella* is one of the most important zoonotic pathogens worldwide. Swine represent typical reservoirs of this bacterium and a frequent source of human infection. Some intrinsic traits make some serovars or strains more virulent than others. Twenty-nine *Salmonella* spp. isolated from pigs belonging to 16 different serovars were analyzed for gastric acid environment resistance, presence of virulence genes (*mgtC*, *rhuM*, *pipB*, *sopB*, *spvRBC*, *gipA*, *sodCI*, *sopE*), antimicrobial resistance and presence of antimicrobial resistance genes (*bla_TEM_*, *bla_PSE-1_*, *aadA1*, *aadA2*, *aphA1*-lab, *strA-strB*, *tetA*, *tetB*, *tetC*, *tetG*, *sul1*, *sul2*, *sul3*). A percentage of 44.83% of strains showed constitutive and inducible gastric acid resistance, whereas 37.93% of strains became resistant only after induction. The genes *sopB*, *pipB* and *mgtC* were the most often detected, with 79.31%, 48.28% and 37.93% of positive strains, respectively. *Salmonella virulence plasmid* genes were detected in a *S*. *enterica* sup. *houtenae* ser. 40:z_4_,z_23_:-strain. Fifteen different virulence profiles were identified: one isolate (ser. Typhimurium) was positive for 6 genes, and 6 isolates (3 ser. Typhimurium, 2 ser. Typhimurium monophasic variant and 1 ser. Choleraesuis) scored positive for 5 genes. None of the isolates resulted resistant to cefotaxime and ciprofloxacin, while all isolates were susceptible to ceftazidime, colistin and gentamycin. Many strains were resistant to sulfonamide (75.86%), tetracycline (51.72%), streptomycin (48.28%) and ampicillin (31.03%). Twenty different resisto-types were identified. Six strains (4 ser. Typhimurium, 1 ser. Derby and 1 ser. Typhimurium monophasic variant) showed the ASSuT profile. Most detected resistance genes *sul2* (34.48%), *tetA* (27.58%) and *strA-strB* (27.58%). Great variability was observed in analyzed strains. *S*. ser. Typhimurium was confirmed as one of the most virulent serovars. This study underlines that swine could be a reservoir and source of pathogenic *Salmonella* strains.

## 1. Introduction

Salmonellosis represents one of the most important zoonosis worldwide, and in 2018 in Europe, *Salmonella enterica* sub *enterica* was the second most common zoonosis agent [1]. In the epidemiology of this bacterium, food acts as the main source of infection and animals as asymptomatic carriers. Many domestic animals could be reservoirs of salmonellae and, in particular, swine are the animals most often infected by this bacterium [2].

To establish infection, salmonellae must exceed the stomach, where the gastric environment represents the first barrier encountered by enteric bacteria. Salmonellae possess the ability to survive the gastric acidity. In particular, for *Salmonella* serovar Typhimurium, two distinct mechanisms to survive low pH were identified: a constitutive/intrinsic acid resistance and an inducible acid resistance, which is activated in acid-shocked cells at pH 4.5 [3]. At the intestinal level, in appropriate conditions, salmonellae could colonize and invade gut epithelium [4]. Almost all *Salmonella* possess the *Salmonella* Pathogenicity Island 1 (SPI1), a genetic element on the chromosome that contains virulence genes, encoding for factors involved in epithelial cells’ invasion [5]. Presence of other SPI or single virulence genes could represent an advantage for bacteria to establish the infection. *Salmonella* Pathogenicity Island 5 (SPI5) encodes for effector proteins, in particular SopB, involved in triggering fluid secretion [6]. The genes *sopE* and *gipA*, transferred by phages, help *Salmonella* to invade host cells and to grow and survive in the Peyer’s patches, respectively [7,8]. Systemic disease can occur in some cases. *Salmonella* Pathogenicity Island 2 (SPI2) is another well-conserved chromosomic element, it allows *Salmonella* to survive inside macrophage and it facilitates spreading through the host body [5]. Other mobile elements could facilitate this event: *Salmonella* Pathogenicity Island 3 (SPI3) and SPI5 encode factors that modify the macrophage environment; the *spv* genes, located on a virulence plasmid, are present in the majority of strains associated with extra-intestinal infections in humans and animals [9]; lastly, *sodCI* is a phage-associated gene that protects *Salmonella* against *oxidative burst* [10].

Antimicrobial resistance is not only a great problem of public health, but it could represent an advantage in *Salmonella* pathogenesis [11]. In recent years, a high percentage of antimicrobial-resistant salmonellae was frequently observed in Europe; in particular, resistance was observed for tetracycline, sulfonamides/sulfamethoxazole and ampicillin. Moreover, an increasing number of multidrug-resistant isolates were recovered [12,13]. In particular, ASSuT profile (ampicillin, streptomycin, sulfonamides, tetracycline) and ACSSuT profile (chloramphenicol, streptomycin, sulfonamides, tetracycline) represent two multidrug resistance profiles typically associated to more pathogenic strains [14]. Antimicrobial resistance genes, in particular the ones related to these specific profiles, could be present on mobile elements like plasmids and integrons. In particular, numerous antimicrobial resistance cassettes (ARC), harboring different combination of antimicrobial resistance genes, were identified in *Salmonella* integrons [15].

This work aims to characterize *Salmonella* spp. isolates from pig for some of their virulence traits. In particular, the strains have been evaluated for: (I) the ability to survive the gastric acid environment with and without low pH induction, (II) the presence of some virulence genes: *mgtC* and *rhuM* located on SPI3, *pipB* and *sopB* located on SPI5, *spvR*, *spvC* and *spvB* located on the *Salmonella virulence plasmid*, *gipA*, *sodCI* and *sopE* located on prophage and (III) the resistance to 22 antimicrobials.

## 2. Materials and Methods

### 2.1. Samples, Bacterial Isolation and Characterization

From January to December 2016, 1480 samples were collected from healthy pigs. In particular, 696 were fecal samples collected from swine of different farms via transrectal swab at slaughterhouse, immediately after jugulation, whereas 784 were organ samples (gut, spleen, liver and mesenteric lymph node) collected at slaughterhouse from 196 animals. All animals are *Large withe* swine, coming from intensive or semi-extensive farms located in Tuscany, Central Italy. All animals were adult, finishing pigs, regularly slaughtered, and no symptoms or lesions were recorded by official veterinarians during ante- and post-mortem examination, respectively.

Samples were kept at 4 °C, transported within 4 h to the Bacteriology Laboratory of the Department of Veterinary Science, University of Pisa, and immediately analyzed. *Salmonella* spp. isolation was carried out as previously described [16]. All isolates were serotyped by the “Istituto Zooprofilattico Sperimentale Lazio e Toscana, sezione di Roma”. *Salmonella* isolates belonging to the same serovars were analyzed with Pulsed Field Gel Electrophoresis (PFGE), as described by other authors [17]. Only one strain for each pulsotype was selected to avoid redundancy results in the subsequent investigations. Nine *Salmonella* strains, belonging to different serovars and pulsotypes, previously isolated from pigs, were also included in the study (1 *S*. ser. Derby, 1 *S*. ser. Mbandaka, 3 *S*. ser. Typhimurium, 1 *S*. ser. Give, 1 *S*. ser. Livingstone, 1 *S*. ser. Infantis and 1 *S*. ser. Choleraesuis).

### 2.2. Resistance to Gastric Acid Environment

To evaluate constitutive and inducible gastric acid resistance, the procedure described by Xia et al. [18] was employed without modifications. The fixed value to consider a strain resistant was 1% of survived cells.

### 2.3. Presence of Virulence Genes

DNA extraction was performed with the DNeasy Blood and Tissue Kit (Qiagen, GmbH, Hilden, Germany) from overnight bacterial cultures. Presence of the following genes was evaluated using primers and protocols reported by other authors: *mgtC*, *rhuM*, *pipB*, *sopB*, *spvR*, *spvC*, *spvB*, *gipA*, *sodCI* and *sopE* [19,20,21,22,23].

### 2.4. Antimicrobial Resistance

Susceptibility to antimicrobials was evaluated with the disc diffusion test on Mueller Hinton Agar (Oxoid, Ltd., Basingstoke, UK), as described in the Clinical and Laboratory Standards Institute (CLSI) manual [24]. The following antibiotics were employed (Oxoid): amoxicillin-clavulanic acid (AMC; 30 µg), ampicillin (AMP; 10 µg), amikacin (AK; 30 µg), cephalothin (KF; 30 µg), cefotaxime (CTX; 30 µg), ceftazidime (CAZ; 30 µg), chloramphenicol (C; 30 µg), ciprofloxacin (CIP; 5 µg), colistin (CT; 10 µg), enrofloxacin (ENR; 5 µg), florphenicol (FFC; 30 µg), gentamycin (CN; 10 µg), kanamycin (K; 30 µg), nalidixic acid (NA; 2 µg), nitrofurantoin (F; 300 µg), streptomycin (S; 10 µg), sulfamethoxazolo-trimethoprim (STX; 25 µg), sulfonamide (S3; 300 µg), tetracycline (TE; 30 µg), tigecycline (TGC; 15 µg), tobramycin (TOB; 10 µg) and trimethoprim (W; 5 µg). The zone diameter interpretive criteria suggested by the CLSI were used [25]. Isolates that resulted resistant to at least one antibiotic in three different antimicrobial classes were considered as Multi-Drug Resistant (MDR) [26].

Presence of genes conferring resistance to ampicillin (*bla_TEM_*, *bla_PSE_*_-1_), aminoglycosides (*aadA1*, *aadA2*, *aphA1*-lab, *strA-strB*), tetracyclines (*tetA*, *tetB*, *tetC*, *tetG*) and sulfonamide (*sul1*, *sul2*, *sul3*) were evaluated with primers and protocols previously reported [27,28,29,30,31]. DNA previously extracted for detection of virulence genes was employed as a template in all single endpoint PCRs.

### 2.5. Statistical Analysis

Data were analyzed with Student’s *t*-test (t), Chi-square (X^2^) test and Fisher’s (F) test. The *t*-test was employed to verify if induction of gastric acid resistance causes a statistically significant difference in increase of survived cells. While, Chi-square (X^2^) and Fisher’s (F) tests were used to evaluate the distribution of genes, ACSSuT and MDR patterns among the different serotypes. Statistical significance threshold was set at a *p*-value ≤ 0.05.

## 3. Results

Overall, 65 *Salmonella* spp. were isolated from analyzed samples. Regarding rectal swabs, 39/696 samples (5.6%) resulted positive, whereas 26 *Salmonella* spp. were collected from 25/784 organs samples (3.19%). In particular, 14 lymph node, 9 gut, 1 spleen and 1 liver samples resulted positive. Two different salmonellae, *S*. ser. Derby and *S*. ser. Typhimurium, were collected from one gut sample. Overall, 22/196 (11.22%) animals were positive, *Salmonella* was isolated from both feces and lymph node of three animals.

The most detected serovar was *S*. ser. Derby, with 43 isolates. Seven isolates belonged to *S*. ser. Kapemba. Two isolates for each of the following serovars were detected: Infantis, Rissen, Bovismorbificans and Typhimurium Monophasic Variant (TMV). Two isolates, both from lymph nodes, resulted *S*. *enterica* sup. *houtenae* ser. 40:z_4_,z_23_:-. One isolate of *S*. ser. Veneziana, *S*. ser. Paratyphi B, *S*. ser. London and *S*. ser. Typhimurium was detected. Finally, one isolate was found belonging to Group O4 (B), but it was not typable.

After PFGE analysis, 29 *Salmonella* spp. strains, including the 9 strains previously isolated, were selected. Table 1 reports information about the chosen strains.

Gastric acid survival challenge revealed that 13/29 strains (44.83%) showed both constitutive and inducible resistance, whereas 11/29 (37.93%) strains survived the gastric acid environment only after induction. For 15/29 (51.72%) isolates, the induction of resistance caused a significant (*p* ≤ 0.05) increase of gastric acid resistance (Table 1). Five out of 29 (17.24%) isolates did not even exhibit resistance after induction. In Table 1, results of the gastric acid survival assay for each of the tested strains are reported.

As concerns the presence of virulence genes, the most detected genes were *sopB*, *pipB* and *mgtC*, with 23/29 (79.31%), 14/29 (48.28%) and 11/29 (37.93%) positive isolates, respectively. Genes *sodC*I, *spvRBC*, *rhuM* and *gipA* were found in less than about 20% of the strains (Table 2). None of the strains showed positivity for gene *sopE*. Four isolates resulted negative for all searched genes. Fourteen different virulence profiles were identified. None of the strains possessed all investigated genes, one *S.* ser. Typhimurium had 6 different genes, and 6 strains were positive for 5 different genes, as reported in Table 1.

No statistical differences were observed among distribution of virulence genes among investigated serotypes. To verify if serotype Typhimurium, including monophasic variant, is more virulent than others, isolates belonging to these serotypes were grouped together and compared with all others. In this case, most virulence genes (*sodCI*, *spvRBC*, *rhuM*, *pipB*, *mgtC*, *gipA*) resulted associated to Typhimurium and TMV, with a statistically significant difference (*p* ≤ 0.05).

A high percentage of antimicrobial resistance was observed for sulfonamide, 22/29 (75.86%) resistant strains, tetracycline, 15/29 (51.72%) resistant strains, and streptomycin, 14/29 (48.28%) resistant strains. All tested salmonellae were susceptible to ceftazidime, gentamycin and colistin sulfate. A high percentage of strains (≥90%) resulted susceptible to ciprofloxacin, amoxicillin/clavulanic acid, trimethoprim, sulfamethoxazole/trimethoprim and chloramphenicol. Table 3 reports the percentage of susceptible, intermediate and resistant strains for each of the antimicrobials employed. Three strains were susceptible to all antimicrobials tested. Twenty different resisto-types were identified (Table 1). MDR was observed in 14/29 (48.27%) of the analyzed isolates (Table 1). Six strains showed the ASSuT (Ampicillin, Streptomycin, Sulfonamide, Tetracycline) profile and one of them was also resistant to chloramphenicol (Table 1).

No statistical differences were observed about the distribution of ASSuT profile and MDR among the different serotypes. Considering Typhimurium and TMV more tightly and comparing isolates belonging to these serotypes with all other isolates, the ASSuT profile and MDR resulted associated to the these serotypes, with a statistically significant difference (*p* ≤ 0.05).

As regards antimicrobial resistance genes, genes more often detected were *sul2*, 10/29 (34.48%) positive isolates, *tetA*, 8/29 (27.58%) positive isolates, *strA-strB*, 8/29 (27.58%) positive isolates, and *bla_TEM_*, 6/29 (20.68%) positive isolates. None of the tested salmonellae resulted positive for *bla_PSE-1_*, *tetC* and *aphA1-lab*. Table 4 reports the distribution of resistance genes among the different serovars. Thirteen out of twenty-two isolates did not present resistance genes, while 6/29 salmonellae showed 3 or more different genes (Table 1).

No statistical differences were observed among distribution of resistance genes among different serotypes. However, grouping isolates belonging to serotypes Typhimurium and TMV together and comparing them with all other serotypes, the genes *tetB*, *tetG*, *bla_TEM_*, *strA-strB*, *sul1* and *sul2* resulted significantly (*p* ≤ 0.05) associated to serotypes Typhimurium and TMV.

## 4. Discussion

In this work, virulence traits of *Salmonella* spp. isolated from swine were evaluated. The acquisition of epidemiological information was not a primary objective of the study; however, some consideration could be carried out. The percentage of positive animals detected was in accordance with other works; in particular, the European Food Safety Authority (EFSA) report showed a prevalence in pigs of 3.5%, ranging from 0% to 10.6%, in Europe in 2016 [32]. Other recent works conducted in Italy and Europe reported *Salmonella* prevalence in pigs ranging from about 10% to 20% [2,33,34]. As expected, lymph nodes represented the best samples for *Salmonella* detection, showing the highest percentage of positive results, however the simultaneous investigation on feces, LN, spleen and liver considerably increased the chances to detect positive animals. *Salmonella* ser. Derby was the most detected serovar. This evidence could be considered predictable, because it was one of the serovars most often detected in swine [1,2,32,33,34]. However, it is interesting to note that some atypical salmonellae were isolated, in particular, *S*. ser. Paratyphi B and *S*. *enterica* sup. *houtenae* ser. 40:z4,z23:-, from lymph nodes.

Stomach represents the primary physical barrier that foodborne pathogens must overcome. Resistance to the gastric acid environment could represent a great advantage. In this study, 44.83% of analyzed strains showed constitutive resistance to the gastric acid environment. Moreover, 37.93% of strains became resistant only after exposure to middle–low pH (5.5). In many cases, the percentage of survived cells greatly improved after induction. In particular, for more than half of isolates, a significant increase of survived cells to Synthetic Gastric Juice (SGJ) was registered after pre-exposure to middle–low pH. In Italy, pig meat is widely employed for the production of fresh sausage, and these products reach low pH values during maturation [35]. This environmental condition could induce gastric acid resistance and promote the transit through the stomach for some strains and represent a great advantage for these foodborne pathogens.

The gene *sopB*, located on SPI5, was detected in most of the analyzed strains (79.31%). It is involved in intestinal cells’ invasion and fluid secretion and it is frequently detected in clinical isolates of *Salmonella* [36]. Also *pipB* is located on SPI5, but it is involved in the survival of salmonellae inside host cells, and it is important for systemic dissemination and establishment of carrier state [37]. A percentage of 48.28% of the analyzed strains possessed *pipB*. The third most detected gene was *mgtC*, with 37.93% of positive isolates. This gene is located on SPI3, it is involved in survival of salmonellae in the phagosome environment and it is commonly found in clinical isolates [38]. The gene *rhuM*, involved in systemic dissemination and located on SPI3, was detected in a low number of isolates (17.24%), always in association with *mgtC*. This gene is not frequently found in *Salmonella* field isolates, as suggested by other authors [39]. *Salmonella virulence plasmid* (*spv*) is involved in systemic dissemination and it is typical of septicemic and more virulent strains [9]. In our study, *spv* was detected in a few isolates (20.69%): in all but one *S*. ser. Typhimurium, in *S*. ser. Bovismorbificans and in *S*. ser. Choleraesuis strains. Surprisingly, it was also detected in *S*. *enterica* sup. *houtenae* ser. 40:z_4_,z_23_:-—salmonellae belonging to subspecies *houtenae* are generally considered low pathogenic and rarely involved in animal and human diseases [1,32]. However, *spv* could sometimes be detected in salmonellae other than subspecies *enterica* [16], and isolation of this strain from lymph nodes confirms its ability to cause systemic infection. The virulence gene *sodCI*, located on a prophage, is involved in protection of *Salmonella* from oxidative burst inside macrophage; this gene, important for systemic dissemination and septicemic disease, is generally found in most virulent strains [10,20]. A percentage of 17.24% of the isolates resulted *sodCI*-positive; in particular, *S*. ser. Bovismorbificans, *S*. ser. Choleraesuis and all but one *S*. ser. Typhimurium. Finally, *gipA* was detected in 3/29 (10.34%) isolates: *S*. ser. Choleraesuis and the 2 *S*. ser. TMV. This gene, involved in invasion of the M cells in the Peyer’s Patch, is not frequently found in *Salmonella* strains [7,40]. None of the analyzed isolates resulted positive for *sopE*. This gene, involved in intestinal invasion and fluid secretion, is more often detected in serovar Enteritidis [40].

Four out of the twenty-nine strains were negative for all the investigated genes (1 *S*. ser. Derby, 1 *S*. ser. Kapemba, 1 *S*. ser. Veneziana and the not typable strain). None of the isolates resulted positive for all 8 targeted genes, but 1 isolate, *S*. ser. Typhimurium, was positive for 6 genes, and 6 isolates, 3 *S*. ser. Typhimurium, 2 *S*. ser. TMV and *S*. ser. Choleraesuis, scored positive for 5 genes. Overall, 15 different virulence profiles were detected, highlighting the great variability that could be encountered in field isolates. Statistical analyses seem to suggest that virulence genes are more often associated to serotypes Typhimurium and TMV, confirming the high virulence and pathogenicity of salmonellae belonging to these serovars. Furthermore, the rare report of the other serovars in humans could be linked to their low virulence. Indeed, without the presence of a passive control system, only clinical cases, that require care or hospitalization, are generally notified in humans. It is possible that many infections by less virulent strains, circulating among animals, occurred in humans, not evolving into clinical disease.

The last characteristic evaluated was antimicrobial resistance. Three molecules, ceftazidime, gentamycin and colistin sulfate, resulted effective against all tested strains. None of the isolates were fully resistant to ciprofloxacin and cefotaxime, which are the clinically most important antimicrobials for treatment of human salmonellosis [12]. The highest percentage of resistance was recorded for sulfonamide, with 75.86% of resistant isolates. Sulfonamide compounds are largely used in farm animals and resistance to *Salmonella* is frequently detected [34,41]. A percentage of 51.72% of strains resulted resistant to tetracycline: this antimicrobial is commonly used in veterinary medicine for treatment of bacterial diseases and in the past years, its effectiveness has been decreasing considerably [12,13,34]. High resistance was also detected for streptomycin: 48.28% and 41.38% of resistant and intermediate strains, respectively. A low level of resistance to the other aminoglycoside compounds tested was encountered, except for kanamycin, with 58.62% of non-susceptible isolates. These data are in agreement with recent reports focused on antimicrobial resistance of salmonellae isolated from swine [34,41]. Low susceptibility was also detected for tigecycline, nitrofurantoin and ampicillin: 96.55%, 44.83% and 41.38% of non-susceptible strains, respectively. Concerning tigecycline, only 24.14% resulted resistant, whereas 72.41% were classified as intermediate. It is a broad-spectrum antimicrobial recently approved for human treatment, however different studies underlined the emergence of resistant isolates among *Salmonella* [40,42]. Use of nitrofurantoin in food-producing animals has been illegal since the early nineties of the last century; however, resistance among *Salmonella* isolates was frequently detected [43]. Finally, different authors reported a worrying increase of ampicillin resistance in *Salmonella* isolates from swine and humans [12,13,34,43]. Only 3/29 strains resulted susceptible to all antimicrobials tested, *S*. ser. Veneziana, *S*. ser. Bovismorbificans and 1 *S*. ser. Derby, while the other strains were resistant to from 1 to 7 different antimicrobials. In particular, 1 strain, *S*. ser. Derby, was resistant to 7 antimicrobials, 2 strains, both belonging to *S*. ser. Typhimurium, were resistant to 6 antimicrobials, and 4 strains, 1 *S*. ser. Give, 1 *S*. ser. London, 1 *S*. ser. Typhimurium and 1 *S*. ser. TMV, resulted resistant to 5 different compounds. Multi-Drug-Resistant isolates were 48.27% of the total, and his value was in line with the last EFSA report on antimicrobial resistance, where the reported percentage of MDR *Salmonella* strains isolates from pigs was 47.4% [13]. ASSuT and ACSSuT profiles, often associated to more virulent strains [14], were detected in five strains, 3 *S*. ser. Typhimurium, 1 *S*. ser. Derby and 1 *S*. ser. TMV, and one, *S*. ser. Typhimurium isolate, respectively.

As expected, considering phenotypic profiles, antimicrobial resistance genes conferring resistance for sulfonamides, aminoglycosides and tetracyclines were the most detected. Twelve out of twenty-nine (41.37%) isolates harbored *sul* genes alone or in combination, 12/29 (41.37%) had at least one aminoglycosides resistance gene, while 11/29 (37.93%) isolates scored positive for one or more *tet* genes. The gene *sul3* was rarely reported in *Salmonella*, in accordance with present data [44,45]. The gene *sul2* resulted more present that *sul1* in analyzed isolates, while other authors reported the two genes equally diffused among *Salmonella* [44,45]. This could be linked to the origin of the samples, indeed, among pigs, it seems *sul2* is more frequently detected than *sul1* [46]. Among aminoglycosides resistance genes, *strA-strB* was the most detected, and this gene was frequently detected in streptomycin-resistant salmonellae. All but one, *S*. ser. Choleraesuis, positive isolates scored positive for only one of the searched aminoglycosides resistance genes [47,48]. *tetA* was confirmed as the more frequent tetracycline resistance gene present among *Salmonella* [47,49,50]. Presence of *bla_TEM_* explained the resistance to Ampicillin in all but 2 resistant isolates. This gene was frequently detected among *Salmonella* [48,49]. Almost all gene-positive isolates were phenotypically resistant to the respective antimicrobials. However, some resistant isolates did not score positive for the searched genes. This is probably related to the presence of other resistance genes in the analyzed salmonellae. Some serotypes, in particular Typhimurium and Typhimurium Monophasic Variant, resulted positive more often for more than one gene. Also, for antimicrobial resistance, ASSuT profile, MDR and resistance genes resulted more associated to serotypes Typhimurium and TMV, observations confirmed by statistical analyses. These findings add other “weapons” to the “arsenal” of these serotypes, and probably contribute to explaining why infection by these serotypes needs particular antimicrobial treatment and sometimes hospitalization, and it stresses the fact that they are more dangerous for human health.

## 5. Conclusions

The obtained results highlight that a great variability exists among *Salmonella* serovars and strains circulating in swine. Although it is not possible to draw up a ranking, some serovars resulted more virulent than others; in particular, the isolates belonging to *S*. ser. Typhimurium exhibited many of the pathogenic traits investigated, and these evidences confirm Typhimurium as one of the most virulent serovars. This study underlines and confirms that many virulent *Salmonella* strains circulate among pigs and that swine represent a source of pathogenic salmonellae for humans.

## Figures and Tables

**Table 1:** Gastric acid resistance, virulence genes and antimicrobial resistance profiles of analyzed *Salmonella* spp. strains.

Isolate	Serotype	Source	Gastric Environment ResistanceMean Value ± s.d. *		Virulence Genes Profile	Antimicrobial Resistance Profile	MDR	Antimicrobial Resistance Genes
			Constitutive	Induced				
**S29**	Derby	I	1.31 ± 0.88	10.15 ± 3.18	*sopB*	TE		
**S34**	Derby	I	4.39 ± 1.87	10.87 ± 2.11 ^†^	*sopB*	S TE S3	•	*tetA aadA1*
**S76**	Derby	L	0.00 ± 0.00	0.51 ± 0.52	*sopB mgtC*	**AMP** KF K **S TE S3** F	•	*bla_TEM_ strA-strB*
**S77**	Mbandaka	I	2.67 ± 1.91	4.64 ± 3.38	*sopB*	TE TGC S3		
**S78**	Typhimurium	I	3.68 ± 2.79	36.50 ± 4.34 ^†^	*spvRBC sopB rhuM pipB mgtC*	NA **AMP S TE** TGC **S3**	•	*tetA bla_TEM_ strA-strB sul2*
**S79**	Give	I	2.35 ± 2.76	8.12 ± 2.26 ^†^	*sopB*	TE TGC S3 W SXT		*strA-strB sul2*
**S80**	Livingstone	L	0.00 ± 0.00	0.47 ± 0.66	*sopB*	TE TGC S3 F	•	*tetA sul2*
**S82**	Infantis	LN	0.00 ± 0.00	0.00 ± 0.00	*sopB pipB*	TE TGC S3		*sul2 sul3*
**S83**	Typhimurium	I	0.60 ± 0.31	0.53 ± 0.61	*sodCI spvRBC sopB rhuM pipB mgtC*	NA **AMP S TE S3**	•	*tetA bla_TEM_ strA-strB sul2*
**S99**	Choleraesuis	L	0.00 ± 0.00	0.00 ± 0.00	*sodCI spvRBC sopB pipB gipA*	S TE S3	•	*tetA aadA2 strA-strB sul2 sul3*
**S100**	Typhimurium	I	1.44 ± 0.60	1.26 ± 1.10	*sodCI sopB rhuM pipB mgtC*	AMP S TE S3	•	*tetB tetG bla_TEM_ strA-strB sul2*
**S169**	Kapemba	I	0.31 ± 0.16	14.39 ± 1.00 ^†^	*mgtC*	S3		
**S177**	Infantis	I	0.67 ± 0.02	3.01 ± 1.08	*sopB pipB*	AMP S S3	•	
**S179**	Derby	I	2.95 ± 1.06	64.20 ± 2.81 ^†^	*sopB*	S3		
**S184**	Kapemba	I	0.72 ± 0.01	23.29 ± 3.98 ^†^		S3		
**S192**	Infantis	Ie	0.10 ± 0.12	17.96 ± 3.25 ^†^	*pipB*	S S3 F	•	*sul2*
**S193**	Derby	I	6.02 ± 5.47	39.44 ± 12.53 ^†^		S3		
**S198**	Veneziana	I	0.91 ± 0.56	29.71 ± 0.16 ^†^				
**S203**	Group 4	I	6.01 ± 5.69	32.71 ± 10.50 ^†^		F		
**S272**	Derby	I	5.61 ± 1.80	43.18 ± 1.24 ^†^	*sopB*			
**S276**	Rissen	I	13.19 ± 3.71	13.81 ± 1.42	*sopB*	TE		*tetA*
**S293**	Bovismorbificans	LN	0.21 ± 0.16	3.85 ± 0.54 ^†^	*sodCI spvRBC sopB pipB*			
**S311**	Paratyphi B	LN	0.66 ± 0.28	6.04 ± 0.21 ^†^	*sopB pipB mgtC*	S		
**S315**	Derby	LN	3.98 ± 1.75	2.54 ± 2.06	*sopB pipB mgtC*	S TOB TE S3	•	*tetA aadA2*
**S317**	London	I	0.03 ± 0.02	6.48 ± 1.38 ^†^	*sopB pipB mgtC*	AMP TE TGC S3 F	•	*aadA2*
**S322**	Typhimurium	I	0.04 ± 0.04	3.98 ± 0.55 ^†^	*sodCI spvRBC sopB pipB mgtC*	**AMP S TE** TGC **S3 C**	•	*tetA tetG aadA2 sul1*
**S324**	40:z_4_,z_23_:-	LN	0.53 ± 0.13	1.32 ± 0.52	*spvRBC sopB*	S AK S3		
**S327**	TMV	I	0.34 ± 0.08	4.64 ± 1.12	*sopB rhuM pipB mgtC gipA*	AMP S S3 F	•	*tetB bla_TEM_ strA-strB sul2*
**S333**	TMV	I	1.55 ± 0.60	4.54 ± 0.61	*sopB rhuM pipB mgtC gipA*	**AMP S TE S3** F	•	*tetB bla_TEM_ strA-strB sul2*

Legend: TMV = Typhimurium Monophasic Variant; I = Intestine, L = Liver, LN = Lymph Nodes; * Percentage of survived cells; s.d. = standard deviation;^†^ the presence of this symbol indicates a statistically significant increase of survived cells after induction; AMP = ampicillin, AK = amikacin, KF = cephalothin, C = chloramphenicol, K = kanamycin, NA = nalidixic acid, F = nitrofurantoin, S = streptomycin, STX = sulfamethoxazolo-trimethoprim, S3 = sulfonamide, TE = tetracycline, TGC = tigecycline, TOB = tobramycin, W trimethoprim. in bold, the ASSuT and ACSSuT profiles; MDR: Multi-Drug Resistant, • = These strains shoved a multidrug resistant profile.

**Table 2:** Distribution of virulence genes among different analyzed *Salmonella* spp. strains.

Serotype (No. of Analyzed Strains)	Virulence Genes							
	*sodCI*	*sopE*	*spvRBC*	*sopB*	*rhuM*	*pipB*	*mgtC*	*gipA*
Bovismorbificans (1)	1	0	1	1	0	1	0	0
Choleraesuis (1)	1	0	1	1	0	1	0	1
Derby (7)	0	0	0	6	0	1	2	0
Give (1)	0	0	0	1	0	0	0	0
Group 4 (1)	0	0	0	0	0	0	0	0
Infantis (3)	0	0	0	2	0	3	0	0
Kapemba (2)	0	0	0	0	0	0	1	0
Livingstone (1)	0	0	0	1	0	0	0	0
London (1)	0	0	0	1	0	1	1	0
Mbandaka (1)	0	0	0	1	0	0	0	0
Paratyphi B (1)	0	0	0	1	0	1	1	0
Rissen (1)	0	0	0	1	0	0	0	0
Typhimurium (4)	3	0	3	4	3	4	4	0
TMV (2)	0	0	0	2	2	2	2	2
Veneziana (1)	0	0	0	0	0	0	0	0
40:z_4_,z_23_:- (1)	0	0	1	1	0	0	0	0
Total (29)	5	0	6	23	5	14	11	3

**Table 3:** Antimicrobial resistance of *Salmonella* spp. strains.

Antimicrobial		Susceptible		Intermediate		Resistant		Non-Susceptible	
		No.	%	No.	%	No.	%	No.	%
Fluoroquinolones	NA	26	89.66	1	3.45	2	6.90	3	10.34
	CIP	27	93.10	2	6.90	0	0.00	2	6.90
	ENR	24	82.76	5	17.24	0	0.00	5	17.24
Penicillins	AMP	17	58.62	3	10.34	9	31.03	12	41.38
	AMC	27	93.10	2	6.90	0	0.00	2	6.90
Cephems (cephalosporins)	CTX	24	82.76	5	17.24	0	0.00	5	17.24
	KF	24	82.76	4	13.79	1	3.45	5	17.24
	CAZ	29	100.00	0	0.00	0	0.00	0	0.00
Aminoglycosides	CN	29	100.00	0	0.00	0	0.00	0	0.00
	K	12	41.38	16	55.17	1	3.45	17	58.62
	S	3	10.34	12	41.38	14	48.28	26	89.66
	AK	26	89.66	2	6.90	1	3.45	3	10.34
	TOB	21	72.41	7	24.14	1	3.45	8	27.59
Tetracyclines	TE	12	41.38	1	3.45	15	51.72	16	57.14
	TGC	1	3.45	21	72.41	7	24.14	28	96.55
Folate pathway inhibitors	S3	6	20.69	1	3.45	22	75.86	23	79.31
	W	28	96.55	0	0.00	1	3.45	1	3.45
	SXT	28	96.55	0	0.00	1	3.45	1	3.45
Others	CT	29	100.00	0	0.00	0	0.00	0	0.00
	F	16	55.17	6	20.69	7	24.14	13	44.83
	C	27	93.10	1	3.45	1	3.45	2	6.90
	FFC	26	89.66	3	10.34	0	0.00	3	10.34

Legend: AMC = amoxicillin-clavulanic acid, AMP = ampicillin, AK = amikacin, KF = cephalothin, CTX = cefotaxime, CAZ = ceftazidime, C = chloramphenicol, CIP = ciprofloxacin, CT = colistin, ENR = enrofloxacin, FFC = florphenicol, CN = gentamycin, K = kanamycin, NA = nalidixic acid, F = nitrofurantoin, S = streptomycin, STX = sulfamethoxazolo-trimethoprim, S3 = sulfonamide, TE = tetracycline, TGC = tigecycline, TOB = tobramycin, W trimethoprim.

**Table 4:** Distribution of antimicrobial resistance genes among analyzed *Salmonella* spp. strains.

Serotype (No. of Analyzed Strains)		Resistance Genes											
	*tetA*	*tetB*	*tetC*	*tetG*	*aadA1*	*aadA2*	*aphA1-lab*	*strA-strB*	*bla_TEM_*	*bla_PSE-1_*	*sul1*	*sul2*	*sul3*
Bovismorbificans (1)	0	0	0	0	0	0	0	0	0	0	0	0	0
Choleraesuis (1)	1	0	0	0	0	1	0	1	0	0	0	1	1
Derby (7)	2	0	0	0	1	1	0	1	1	0	0	0	0
Give (1)	0	0	0	0	0	0	0	1	0	0	0	1	0
Group 4 (1)	0	0	0	0	0	0	0	0	0	0	0	0	0
Infantis (3)	0	0	0	0	0	0	0	0	0	0	0	2	1
Kapemba (2)	0	0	0	0	0	0	0	0	0	0	0	0	0
Livingstone (1)	1	0	0	0	0	0	0	0	0	0	0	1	0
London (1)	0	0	0	0	0	1	0	0	0	0	0	0	0
Mbandaka (1)	0	0	0	0	0	0	0	0	0	0	0	0	0
Paratyphi B (1)	0	0	0	0	0	0	0	0	0	0	0	0	0
Rissen (1)	1	0	0	0	0	0	0	0	0	0	0	0	0
Typhimurium (4)	3	1	0	2	0	1	0	3	3	0	1	3	0
TMV (2)	0	2	0	0	0	0	0	2	2	0	0	2	0
Veneziana (1)	0	0	0	0	0	0	0	0	0	0	0	0	0
40:z_4_,z_23_:- (1)	0	0	0	0	0	0	0	0	0	0	0	0	0
Total (29)	8	3	0	2	1	4	0	8	6	0	1	10	2
